# Identification of *NSP3* (*SH2D3C*) as a Prognostic Biomarker of Tumor Progression and Immune Evasion for Lung Cancer and Evaluation of Organosulfur Compounds from *Allium sativum* L. as Therapeutic Candidates

**DOI:** 10.3390/biomedicines9111582

**Published:** 2021-10-30

**Authors:** Yuan-Chieh Yeh, Bashir Lawal, Michael Hsiao, Tse-Hung Huang, Chi-Ying F. Huang

**Affiliations:** 1 Program in Molecular Medicine, College of Life Sciences, National Yang Ming Chiao Tung University, Taipei 11221, Taiwan; b9005030@gmail.com; 2 Department of Traditional Chinese Medicine, Chang Gung Memorial Hospital, Keelung 20401, Taiwan; 3 PhD Program for Cancer Molecular Biology and Drug Discovery, College of Medical Science and Technology, Taipei Medical University, Taipei 11031, Taiwan; bashirlawal12@gmail.com; 4 Graduate Institute of Cancer Biology & Drug Discovery, College of Medical Science and Technology, Taipei Medical University, Taipei 11031, Taiwan; 5 Genomics Research Center, Academia Sinica, Taipei 115201, Taiwan; mhsiao@gate.sinica.edu.tw; 6 School of Traditional Chinese Medicine, Chang Gung University, Kweishan, Taoyuan 333, Taiwan; 7 School of Nursing, National Taipei University of Nursing and Health Sciences, Taipei 112, Taiwan; 8 Graduate Institute of Health Industry Technology, Chang Gung University of Science and Technology, Taoyuan 333, Taiwan; 9 Research Center for Chinese Herbal Medicine, Chang Gung University of Science and Technology, Taoyuan 333, Taiwan; 10 Department & Graduate Institute of Chemical Engineering & Graduate Institute of Biochemical Engineering, Ming Chi University of Technology, New Taipei City 243, Taiwan; 11 Institute of Biopharmaceutical Sciences, College of Pharmaceutical Sciences, National Yang Ming Chiao Tung University, Taipei 11221, Taiwan; 12 Department of Biochemistry, School of Medicine, Kaohsiung Medical University, Kaohsiung 80708, Taiwan

**Keywords:** *NSP3 (SH2D3C)*, NSCLC, tumor microenvironment, immune infiltrations, dysfunctional T-cell phenotypes, in silico study, pharmacokinetics, organosulfur compounds

## Abstract

The novel SH2-containing protein 3 (*NSP3*) is an oncogenic molecule that has been concomitantly associated with T cell trafficking. However, its oncological role in lung cancer and whether it plays a role in modulating the tumor immune microenvironment is not properly understood. In the present in silico study, we demonstrated that *NSP3 (SH2D3C)* is associated with advanced stage and poor prognoses of lung cancer cohorts. Genetic alterations of *NSP3 (SH2D3C*) co-occurred inversely with Epidermal Growth Factor Receptor (*EGFR*) alterations and elicited its pathological role via modulation of various components of the immune and inflammatory pathways in lung cancer. Our correlation analysis suggested that *NSP3 (SH2D3C)* promotes tumor immune evasion via dysfunctional T-cell phenotypes and T-cell exclusion mechanisms in lung cancer patients. *NSP3 (SH2D3C)* demonstrated a high predictive value and association with therapy resistance in lung cancer, hence serving as an attractive target for therapy exploration. We evaluated the in silico drug-likeness and *NSP3 (SH2D3C)* target efficacy of six organosulfur small molecules from *Allium sativum* using a molecular docking study. We found that the six organosulfur compounds demonstrated selective cytotoxic potential against cancer cell lines and good predictions for ADMET properties, drug-likeness, and safety profile. E-ajoene, alliin, diallyl sulfide, 2-vinyl-4H-1,3-dithiin, allicin, and S-allyl-cysteine docked well into the *NSP3 (SH2D3C)*-binding cavity with binding affinities ranging from −3.5~−6.70 Ă and random forest (RF) scores ranging from 4.31~5.26 pKd. In conclusion, our study revealed that *NSP3* is an important onco-immunological biomarker encompassing the tumor microenvironment, disease staging and prognosis in lung cancer and could serve as an attractive target for cancer therapy. The organosulfur compounds from *A. sativum* have molecular properties to efficiently interact with the binding site of *NSP3* and are currently under vigorous preclinical study in our laboratory.

## 1. Introduction

Lung cancer is the second most commonly diagnosed cancer, with an estimated 2.2 million new cases (11.4%) worldwide, and remains the leading cause of cancer deaths, with an estimated 1.8 million deaths (18%) in 2020 [1]. With higher incidences and mortality rates in men than in women, the global burden of lung cancer is ranked first in men, whereas, in women, it ranks second behind breast cancer [1,2]. This pattern is largely attributed to air pollution and increased tobacco consumption [3,4]. Histologically, the majority (80%) of lung cancers are non-small-cell lung cancer (NSCLC) while the remaining 20% are small-cell lung cancer (SCLC) [5,6]. NSCLC is further divided into three pathological subtypes of lung adenocarcinoma (LUAD), squamous cell carcinoma, and large-cell carcinoma, with LUAD being the most common subtype [7,8]. Despite advances in treatment strategies such as chemotherapy, immunotherapy, radiotherapy, and surgery, the prognosis of lung cancer is still disappointing with fewer than 20% of patients reaching an overall 5-year survival period [9,10].

A tumor-driven immune imbalance plays an important role in tumor initiation and progression. The tumor microenvironment (TME) is a complex system that encompasses various stroma cell types, immune cell types, and other extracellular components [11]. Interactions among components of the TME play pivotal roles in tumor occurrence and progression [12]. In addition, the efficacy of immune checkpoint blockades is regulated by the interplay between components of the TME [13,14]. It was initially thought that tumor immune infiltration was strictly associated with tumor-induced immune suppression. However, increasing evidence suggested that both genetic and epigenetic alterations within the TME mediate tumor immune evasion and tumor progression via distinct mechanisms involving T-cell anergy and dysfunctional T-cell phenotypes in various cancer types. In addition, tumor infiltrations of immunosuppressive cells, such as cancer-associated fibroblasts (CAFs), M2-macrophages, and regulatory T (Treg) cells, are known to mediate tumor evasion of immune cells by inhibiting the activities of cytotoxic T cells via a T-cell exclusion mechanism [15,16]. Previous studies reported that CAFs induce the formation of a pre-metastatic niche and increase metastasis in lung cancer patients [17]. CAFs decrease drug sensitivity by blocking the delivery of drugs and thus contribute to the poor prognosis of cancer patients [18,19].

The novel SH2-containing protein 3 (NSP3) is an oncogenic molecule that regulates T cell receptor signaling [20]. It acts as an adapter protein that mediates cell signaling pathways involved in cellular functions such as cell adhesion and migration, tissue organization, and the regulation of the immune response [20,21,22]. It thus could be an attractive target for the development of therapeutic strategies for treating cancer.

In drug discovery pipelines, natural products, particularly medicinal plants, serve as an important source of natural therapeutic agents for treating several diseases [23,24,25,26]. Garlic (*Allium sativum* L. of the Amaryllidaceae family) is an aromatic herbaceous annual spice with various biological activities [27,28,29,30,31]. It contains hundreds of phytochemicals, among which sulfur-containing compounds are of therapeutic interest in the field of oncology [30,32,33,34,35,36,37,38,39]. Previous studies appraised the uses of in silico approaches to assessing biomarkers and the prognostic relevance of genes and gene signatures in a diseased condition based on the available clinical information of cohorts, and also aid in identifying drug-like molecules for targeted therapy [40,41,42]. In this study, we used a bioinformatics approach to evaluate the effect of *NSP3* expression as well as genetic and epigenetic alterations on the TME with respect to tumor immune evasion mechanisms and prognoses in lung cancer cohorts. In addition, we reported the drug-likeness, pharmacokinetics (PKs), toxicity, and potential therapeutic properties of some sulfur-containing compounds from *A. sativum.* Lastly, we also demonstrated the potential of the compounds to regulate the activity of *NSP3* using molecular docking studies of receptor−ligand interactions.

## 2. Materials and Methods

### 2.1. Differential Expression Analysis of NSP3 (SH2D3C) in Lung Cancer Cohorts

We used the Tumor IMmune Estimation Resource (TIMER2.0) algorithm (http://timer.cistrome.org/ accessed on 23 July 2021) [43], and the Gene Expression Profiling Interactive Analysis (GEPIA) database (http://gepia.cancer-pku.cn/ accessed on 23 July 2021) [44] for the differential expression analysis of *NSP3* (*SH2D3C*) in tumor vs. normal tissues from various cancer types of The Cancer Genome Atlas (TCGA) database. In addition, we used the TNMplot server [45] to conduct a differential gene expression analysis of *NSP3* (*SH2D3C*) between the pathological free (normal), primary cancer, and metastatic lung cancer tissues.

### 2.2. Prognostic Analysis of NSP3 (SH2D3C) in Lung Cancer Cohorts

To analyze the prognostic value of *NSP3*, the expression level and survival information of lung cancer cohorts from GEO, EGA, and TCGA were integrated via PostgreSQL server. We split the patient samples into high and low expression levels based on the quantile expressions levels of the gene. The survival differences between the two groups was visualized using the Kaplan−Meier survival analysis (https://kmplot.com/analysis/ accessed on 21 August 2021) [46]. The hazard ratio with 95% confidence intervals and logrank *p* value are calculated.

### 2.3. Analysis of NSP3 (SH2D3C) Genetic Alterations Frequency and Co-occurrence in Lung Cancer Cohorts

We explore the cancer genomic data set through the cBioPortal tool (http://www.cbioportal.org/ accessed on 20 August 2021) [47,48] to analyze the types and frequencies of *NSP3* genetic alterations in lung cancer cohorts. The survival differences between cohorts with and without alterations in *NSP3* (*SH2D3C*) genome was also analyzed through the server and visualized using the Kaplan−Meier plots. In addition, we analyzed the gene mutation co-occurrence patterns between *NSP3* (*SH2D3C*) and other genes in TCGA lung cancer cohorts. Alteration co-occurrence was considered significant only at *p* < 0.05 and *q* < 0.05.

### 2.4. Analysis of the SH2D3C Association with Immune and Immunosuppressive Cell Infiltration in Lung Cancer

We explore the immune module of the TIMER algorithm (http://timer.comp-genomics.org/ accessed on 5 August 2021) to comprehensively analyze correlations between *NSP3* (*SH2D3C*) expressions and tumor infiltration levels of six immune cell types (B cells, cluster of differentiation-positive (CD8^+^) T cells, CD4^+^ T cells, macrophages, neutrophils, and dendritic cells (DCs)) and immunosuppressive cells (cancer-associated fibroblasts (CAFs), T reg cells, and tumor-associated macrophages (TAMs)) in TCGA cohorts of lung cancer. We used the Tumor Immune Dysfunction and Exclusion (TIDE) (http://tide.dfci.harvard.edu accessed on 3 August 2021) tools [49] to analyze correlations of the expression, methylation, and copy number alterations (CNAs) of *NSP3* (*SH2D3C*) with cytotoxic T lymphocyte (CTL) infiltration, dysfunctional T-cell phenotypes, and T-cell exclusion phenotypes in lung cancer cohorts.

### 2.5. SH2D3C Interaction Network, Functional Enrichment, and Disease-Specific Associations Analysis

The protein−protein (PPI) interaction network of *NSP3* was constructed and analyzed via the search tool for retrieval of interacting genes/proteins (STRING) server (http://string-db.org/, v10.5 accessed on 21 July 2021) [50]. The official gene symbols of *NSP3* were loaded to the single protein modules of the server and analyzed for both predictive and known interactions in *Homo sapiens* under the confidence search of 0.70 and at *p* < 0.05. In addition, the Kyoto Encyclopedia of Genes and Genomes (KEGG) pathways and gene ontology (GO) of the biological process enrichment of the network was downloaded via the server and was further confirmed using the Enrichr platform, an online gene set enrichment analysis (GSEA) server [51,52]. The enrichment plots were generated using the ImageGP (http://www.ehbio.com/ImageGP/ accessed on 27 August 2021) platform. Analysis and visualization of the gene−gene interaction (GGI) network of the *NSP3* was performed through the GeneMANIA platform, a real-time multiple association network integration algorithm for predicting gene functions [53]. The gene disease-specific associations of *NSP3* was analyzed at the confident levels of 0.4 by using the OPENTARGET platform (https://www.targetvalidation.org/ accessed on 27 August 2021), a bio-web algorithm that integrates genetic, omic, and chemical data to identify the involvement of genes in diseases and aid systematic drug target identification and prioritization [54].

### 2.6. SH2D3 Knockdown (shSH2D3C) Efficacy and Gene Expression Correlation Analysis in NSCLC

We evaluated the efficacy of *SH2D3* knockdown (*shSH2D3C*) in association with the expression of a number gene. A total of 58 samples are classified into two groups by the median value of *shSH2D3C* and target genes. Results were presented in the form of predictivity and descriptivity. The predictivity is defined as the fold change (FC) of *shSH2D3C* efficacy between samples of high and low expression of the gene. Descriptivity is defined as the FC of gene expression between samples of high and low *shSH2D3C* efficacy.

### 2.7. In Silico Analysis of the Druglikeness, Pharmacokinetics, and Pharmacology of Some Organosulfur Small Molecules from Allium Sativum

We analyzed the drug-likeness, pharmacokinetics, and medicinal chemistry of six organosulfur small molecules from *A. sativum* (E-ajoene, alliin, diallyl sulfide, 2-vinyl-4H-1,3-dithiin, allicin and **S**-allyl-cysteine) using the SwissADME algorithm [55]. We used the blood−brain barrier (BBB) Prediction Server (https://www.cbligand.org/BBB/ accessed on 21 July 2021), which operates based on the support vector machine (SVM) and LiCABEDS algorithms to analyze the BBB permeation ability of the compounds [56]. We evaluated the in silico cytotoxicity of the compounds against the cancer cell lines and normal cell lines by using the Cell Line Cytotoxicity Predictor (CLC-Pred) modules of the computer-aided Prediction of Biological Activity Spectra (PASS) web resources [57] created based on the training set of data on cytotoxicity retrieved from ChEMBLdb (version 23).

### 2.8. In Silico Acute Toxicity Analysis of the Organosulfur Compounds

We predicted the 50% lethal dose (LD_50_) of the compounds for the different routes of administrations (intraperitoneal, intravenous, oral, and subcutaneous) in rats using the GUSAR software for quantitative structure-activity relationship (QSAR)/quantitative structure-property relationship (QSPR) modeling [58]. The GUSAR software was developed based on training sets of data from the SYMYX MDL Toxicity Database, consisting of approximately 10^4^ chemical structures with data on acute rat toxicity represented by the LD_50_ values (log10 (mmol/kg)). In addition, we also evaluated the ecotoxicity properties of the compounds using the bioaccumulation factor Log10(BCF), *Daphnia magna* 50% lethal concentration (LC_50_) -Log10(mol/L), Fathead Minnow LC_50_ Log10(mmol/L), and *Tetrahymena pyriformis* 50% inhibitory growth concentration (IGC_50_) -log10(mol/L) as indicators.

### 2.9. Molecular Docking of Receptor−Ligand Interaction between SH2D3C and the Organosulfur Compound

The crystal structures of *NSP3* (PDB: 6W6Y) were downloaded from the Protein Data Bank (PDB) (https://www.rcsb.org/ accessed on 15 August 2021) in PDB file format and subsequently converted into the Auto Dock PDBQT format using AutoDock Vina (ver. 0.8, Scripps Research Institute, La Jolla, CA, USA) [59]. Three-dimensional (3D) molecular ball-and-stick models of some organosulfur compounds from *A. sativum* (alliin, allicin, E-ajoene, Z-ajoene, 2-vinyl-4H-1,3-dithiin, diallyl sulfide, and allyl methyl sulfide) were drawn using the Avogadro molecular builder and visualization tool ver. 1.XX and subsequently converted to PDB format using the PyMOL tool and then to PDBQT format using AutoDock Vina. The receptor was prepared by pre-docking removal of water (H_2_0) molecules and the addition of hydrogen atoms (polar only) and Kolman charges [60,61]. Molecular docking was performed using AutoDock Vina as described previously [40,60,62,63]. Docking outcomes were visualized using the Discovery Studio Visualizer ver. 19.1.0.18287 (BIOVIA, San Diego, CA, USA) [64].

### 2.10. Data Analysis

Spearman’s rank correlations were used to assess correlations between *NSP3* expression and infiltration of immunosuppressive cells. A KM curve was employed to visualize survival differences between lung cancer cohorts with high and those with low expression levels of *NSP3 (SH2D3C)*. The statistical significance of the differential expression of *NSP3* between lung cancer tumor and adjacent normal tissue was evaluated using the Wilcoxon test. Statistical significance was indicated by * *p* < 0.05; ** *p* < 0.01; *** *p* < 0.001.

## 3. Results

### 3.1. NSP3 (SH2D3C) Is Associated with Advance Stage and Poor Prognoses of Lung Cancer Cohorts

**Figure 1:**
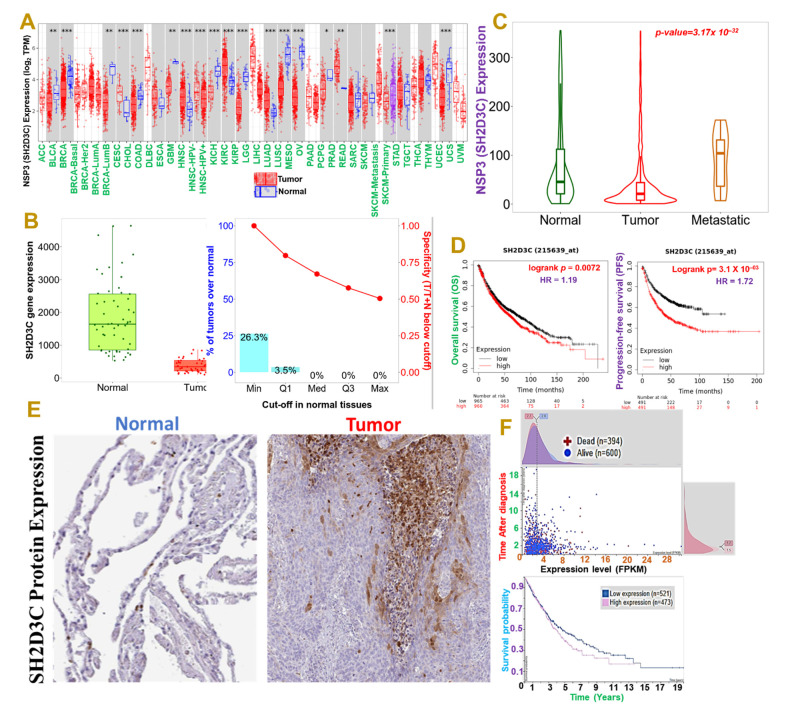
Non-structural protein 3 (*NSP3; SH2D3C*) is associated with advanced stages and poor prognoses of lung cancer cohorts. (**A**) Box plots showing differential expression profile of *NSP3 (SH2D3C)* between tumor and adjacent normal tissue across all TCGA cancer types and (**B**) between tumor and adjacent normal tissues in lung cancer cohort. (**C**) Violine plots showing differential expression profile of *NSP3 (SH2D3C)* between normal tissue, tumor, and metastatic lung cancer patients. (**D**) Kaplan–Meier plot of the overall and progressive-free survival between lung adenocarcinoma patients with low and high expression levels of *NSP3 (SH2D3C*). (**E**) Representative immunohistochemistry staining of *SH2D3C* between lung cancer tumor and pathological free tissue in the Human Protein Atlas (HPA) database. (**F**) Interactive survival scatter plots and Kaplan–Meier plot of the survival probability between high and low mRNA expression levels in lung cancer cohorts. * *p* < 0.05; ** *p* < 0.01; *** *p* < 0.001.

Our differential expression profile of *NSP3 (SH2D3C)* between tumor and adjacent normal tissue revealed that *NSP3 (SH2D3C)* has deregulatory expression in tumor samples of TCGA cancer types (Figure 1A). However, our focus is on lung cancer, and we found that *NSP3 (SH2D3C)* is expressed at a lower level in lung cancer tumors than the adjacent normal tissue (Figure 1B). On a contrary note, we found that cohorts with metastatic lung cancer exhibited higher *NSP3 (SH2D3C)* expression levels than the primary tumor (Figure 1C). Our survival analysis also revealed that patients with higher gene expression levels exhibited a shorter overall and progressive free survival duration than cohorts with lower expression levels (Figure 1D). Furthermore, we queried the differential protein expression levels of *NSP3 (SH2D3C)* between lung cancer tissue and disease-free tissue. Interestingly, we found that lung cancer tumors exhibited higher intensity of *NSP3 (SH2D3C*) than the pathological free tissue (Figure 1E). Moreover, cohorts with high mRNA expression levels of *NSP3* (*SH2D3C*) exhibited shorter survival duration than cohorts with low mRNA expression levels (Figure 1F). Collectively, our results suggest that *NSP3 (SH2D3C)* is associated with the advanced stage and poor prognosis of lung cancer and thus could serve as a prognostic biomarker of lung cancer progression and follow-up.

### 3.2. Genetic Alterations of NSP3 (SH2D3C) Are Associated with Poorer Prognosis and Inversely Associated with EGFR Alterations in Lung Cancer Patients

**Figure 2:**
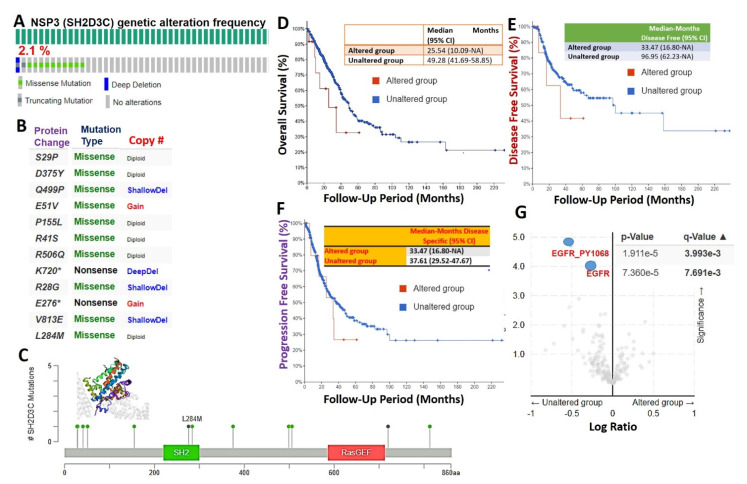
Genetic alterations of non-structural protein 3 (*NSP3; SH2D3C*) are associated with poorer prognosis and inversely associated with Epidermal Growth Factor Receptor (*EGFR*) alterations in lung cancer patients. Genetic alteration profile of *NSP3 (SH2D3C)* in lung adenocarcinoma. (**A**) Prevalence and distribution of *NSP3 (SH2D3C*) genetic alterations in lung adenocarcinoma cohort. (**B**) Specific mutation types of *NSP3 (SH2D3C*) in lung adenocarcinoma cohorts. (**C**) Lollipop plot of *NSP3 (SH2D3C)* mutation location in lung adenocarcinoma cohort across the cBioPortal for Cancer Genomics dataset. Mutations are color-coded as missense, truncating, and in-frame mutations. (**D**) Kaplan–Meier plot of the (**D**) shorter overall survival (**E**), disease-free survival, and (**F**) progression-free survival between lung cancer cohorts with genetically altered and cohorts without altered *NSP3 (SH2D3C).* (**G**) Heat map of *NSP3 (SH2D3C*) mutation co-occurrence in lung cancer patients.

We used the cBioPortal database to query the genetic alteration profile of *NSP3 (SH2D3C)* in 566 lung adenocarcinoma cases (TCGA, PanCancer Atlas) and found that 2.1% of the cohorts harbored genetically altered *NSP3 (SH2D3C)* (Figure 2A). However, mutation and multiple alterations were the only genetic alterations of *NSP3 (SH2D3C)* in lung adenocarcinoma cohorts (Figure 2B). Specific mutation profiling indicated that out of the total *NSP3 (SH2D3C)* mutation in the database, 83.33% were missense (Figure 2B, C) and only a few truncating mutations occur, while no in-frame or fusion mutations were recorded (Figure 2C). Furthermore, we found that the genetic alterations of *NSP3 (SH2D3C)* are associated with shorter overall survival (Figure 2D), disease-free survival (Figure 2E), and progression-free survival (Figure 2F) of the cohorts. We queried the co-occurrence of *NSP3 (SH2D3C)* alterations with other genetic alterations and found that *NSP3 (SH2D3C)* alteration is inversely associated with Epidermal Growth Factor Receptor (*EGFR*) alterations in lung cancer, i.e., lung cancer patients with *NSP3 (SH2D3C)* alteration are devoid of EGFR alterations (Figure 2G).

### 3.3. NSP3 Elicits Its Pathological Role via Modulation of Various Components of the Immune and Inflammatory Pathways in Lung Cancer

**Figure 3:**
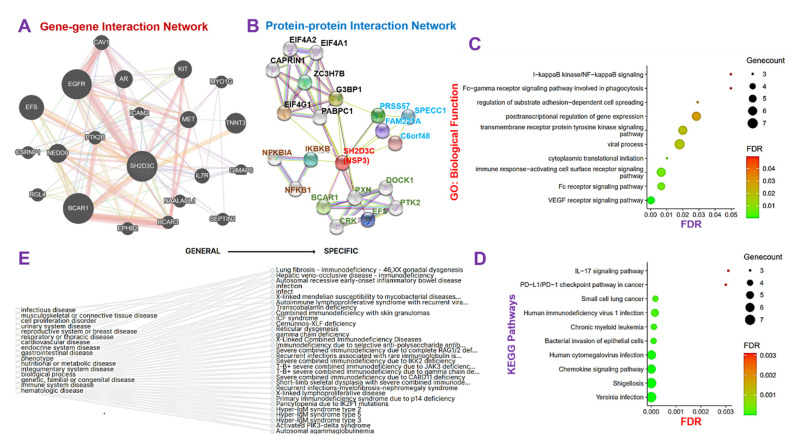
Non-structural protein 3 (*NSP3; SH2D3C*) elicits its pathological role via modulation of various components of the immune and inflammatory pathways in lung cancer. (**A**) Gene−gene interaction (GGI) and (**B**) protein−protein interaction (PPI) network of *NSP3*. (**C**) The Kyoto Encyclopedia of Genes and Genomes and (**D**) G.O. biological function enrichments of the *NSP3* network proteins. (**E**) Gene-diseases associated tree plot of *NSP3*.

The GGI network revealed that the *NSP3* gene interacts with a number of oncogenic, immune, and inflammatory genes including *AR, BCAR1, BCAR3, CAV1, CSRNP1, EFS, EGFR, EPHB2, GIMAP6, ICAM2, IL7R, KIT, MET, MYO1G, NAALADL1, NEDD9, PTK2B, RGL4, SEPTIN1,* and *TNNT* (Figure 3A). Similarly, the PPI network analysis revealed 21 nodes, 48 edges, an average local clustering coefficient of 0.747 and a PPI enrichment *p*-value of 0.000159 (Figure 3B). The nodes with the highest degree in the network included SH2D3C (11), *G3BP1* (8), *EIF4G1* (7), *PABPC1* (7), *BCAR1* (5), *CAPRIN1* (5), *EIF4A1* (5), and *EIF4A2* (5) (Figure 3B). To analyze the functional enrichment of the gene network, we examined the GO biological processes and KEGG pathways, and our results revealed the biological enrichment of the vascular endothelial growth factor receptor (*VEGFR*) signaling pathway, immune response-activating cell surface receptor signaling, Fc receptor signaling pathway, cytoplasmic translational initiation, viral process, transmembrane receptor protein tyrosine kinase signaling pathway, post-transcriptional regulation of gene expression, regulation of substrate adhesion-dependent cell spreading, I-kappaB kinase/NF-kappaB signaling, and Fc-gamma receptor signaling pathway involved in phagocytosis (Figure 3C), while the KEGG pathway analysis revealed enrichment of Yersinia infection, shigellosis, chemokine signaling pathway, human cytomegalovirus infection, bacterial invasion of epithelial cells, chronic myeloid leukemia, human immunodeficiency virus 1 infection, and small cell lung cancer (Figure 3D). Collectively, our results suggested that *NSP3* is involved in the pathology of bacterial and viral infections, immune and inflammatory diseases, and cell proliferation and lung cancer development.

### 3.4. NSP3 (SH2D3C) Promotes Tumor-Immune Evasion via Dysfunctional T-Cell Phenotypes and T-Cell Exclusion Mechanism in Lung Cancer Patients

**Figure 4:**
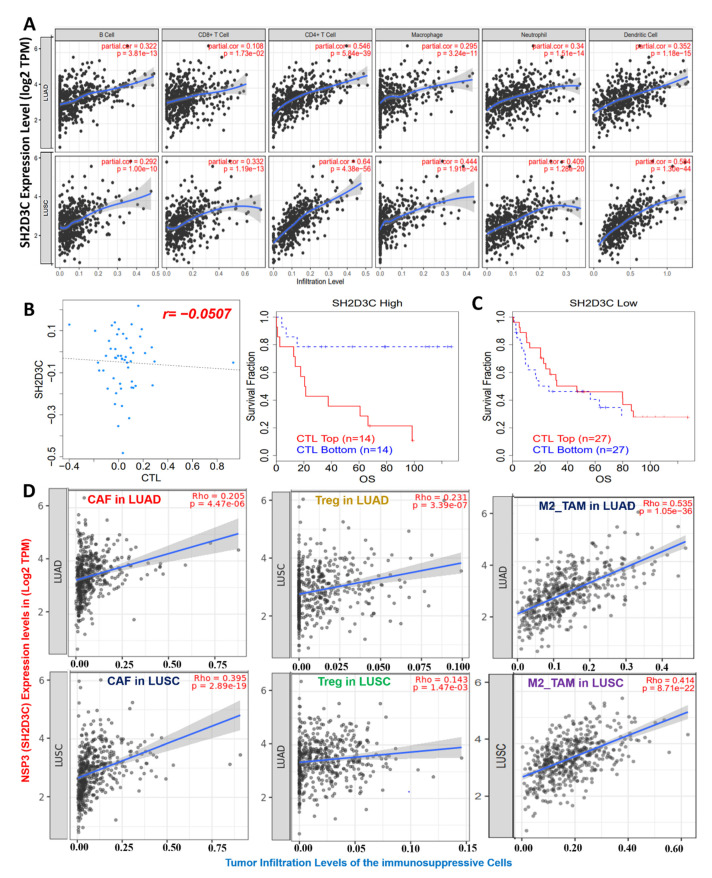
Non-structural protein 3 (*NSP3; SH2D3C*) promotes dysfunctional T-cell phenotypes in lung cancer patients. (**A**) Scatterplot showing correlations between *NSP3 (SH2D3C)* expression and infiltration of various immune cells in lung adenocarcinoma (LUAD) and lung squamous cell carcinoma (LUSC). (**B**) Correlation analysis between *NSP3 (SH2D3C)* expression and cytotoxic T lymphocyte (CTL) levels in lung cancer patients. (**C**) Kaplan–Meier plot of the overall survival of lung cancer patients with different *NSP3 (SH2D3C)* and CTL levels. (**D**) Boxplot showing *NSP3 (SH2D3C)* expression correlations with infiltration levels of immunosuppressive cells, including cancer-associated fibroblasts (CAFs), M2 macrophages, and regulatory T cells in LUAD patients.

We analyzed the relationship of *NSP3 (SH2D3C)* expression with tumor infiltration by immune cells and found that *NSP3 (SH2D3C)* expression showed strong correlations with the infiltration of six immune cell types, including the B cells (*r* = 0.292–0.3220 all *p* < 0.1 × 10^−10^), CD8^+^ T cells (*r* = 0.18–0.33 all *p* < 0.05), CD4^+^ T cells (*r* = 0.54–0.64 all *p* < 0.1 × 10^−30^), M1-macrophages (*r* = 0.295–0.44 all *p* < 0.1 × 10^−10^), neutrophils (*r* = 0.34–0.40 all *p* < 0.1 × 10^−10^), and DCs (*r* = 0.35–0.59 all *p* < 0.1 × 10^−10^) (Figure 4A). Interestingly, we found that *NSP3 (SH2D3C)* expression was inversely associated with CTL levels in lung cancer patients (Figure 4B). In addition, we found that a higher expression profile of *NSP3 (SH2D3C)* in cohorts with higher CTL levels exhibited a shorter survival period, while a lower expression profile of *NSP3 (SH2D3C)* in cohorts with higher CTL levels exhibited a longer survival period, suggesting a correlation with dysfunctional T-cell phenotypes (Figure 4C). Furthermore, we queried associations of *NSP3 (SH2D3C)* expressions with infiltration levels of immunosuppressive cells and found that *NSP3 (SH2D3C)* expression was positively correlated with tumor infiltration of CAFs (*r* = 0.205–0.395 all *p* < 0.1 × 10^−5^), T reg cells (*r* = 0.231–0.143 all *p* < 0.05), and M2 macrophages (*r* = 0.535–0.414 all *p* < 0.1 × 10^−20^) in LUAD and LUSC, respectively (Figure 4D). Altogether, our findings strongly suggest that *NSP3 (SH2D3C)* promotes tumor immune evasion via dysfunctional T-cell phenotypes and T-cell exclusion mechanisms in lung cancer patients.

### 3.5. NSP3 (SH2D3C) Methylation and Copy Number Alterations (CNA) Are Associated with Infiltration of Immune Cells and Poorer Prognosis of Lung Cancer Cohorts

**Figure 5:**
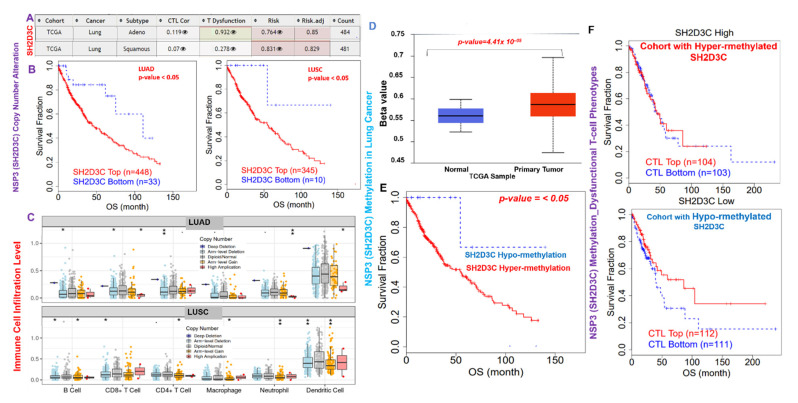
Non-structural protein 3 (*NSP3; SH2D3C*) methylation and copy number alterations (CNA) are associated with infiltration of immune cells and poorer prognosis of lung cancer cohorts. (**A**) Heatmap plot of the *NSP3 (SH2D3C)* expression and T-cell profile in LUAD and LUSC cohorts. (**B**) Kaplan–Meier plot of the overall survival differences between high and low CNA of *NSP3 (SH2D3C)* in LUAD and LUSC cohorts. (**C**) Boxplot showing the association between different somatic copy number alterations and infiltration of various immune cells in LUAD and LUSC. (**D**) Boxplot showing differential *NSP3 (SH2D3C)* methylation between lung cancer tumors and adjacent normal tissue. (**E**) Kaplan–Meier plot of the overall survival differences between lung cancer cohorts with high and low methylation levels of *NSP3 (SH2D3C)*. (**F**) Kaplan–Meier plot of the overall survival of lung cancer patients with different *NSP3 (SH2D3C)* methylation status and CTL levels. The “eye” sign represent the hidden view of the CTL, dysfunctional and risk plots of the cohorts.

We evaluated the effect of CNA and methylation of *NSP3 (SH2D3C*) in lung cancer (Figure 5A–F), and the results revealed that *NSP3 (SH2D3C)* is hypermethylated in tumors of lung cancer patients compared to the normal tissue (Figure 5D). In addition, lung cancer patients with high methylation levels of *NSP3 (SH2D3C)* exhibited shorter survival duration than cohorts with low methylation levels (Figure 5E). Analysis of CTL level and methylation status of *NSP3 (SH2D3C*) revealed that cohorts with high levels of *NSP3 (SH2D3C)* exhibited dysfunctional T-cell phenotypes and poor prognosis (Figure 5F). Nevertheless, we found that the CNA of *NSP3 (SH2D3C)* are a risk factor for LUAD and LUSC cohorts (Figure 5A). Analysis of the patients’ survival revealed that both LUAD and LUSC patients with CNA of *NSP3 (SH2D3C)* exhibited shorter overall survival duration than the cohort with low CNA (Figure 5B). In addition, we found that SCNA of *NSP3 (SH2D3C*) including deep deletion, arm-level deletion, arm-level gain, and high amplification also mediated the infiltrations of the tumor-immune cells in both LUAD and LUSC (Figure 5C). Collectively, these findings prove that hyper-methylation and copy number alterations of *NSP3 (SH2D3C)* are associated with infiltration of immune cells and poorer prognosis of lung cancer cohorts.

### 3.6. SH2D3C Is Associated with Therapy Resistance in NSCLC

**Figure 6:**
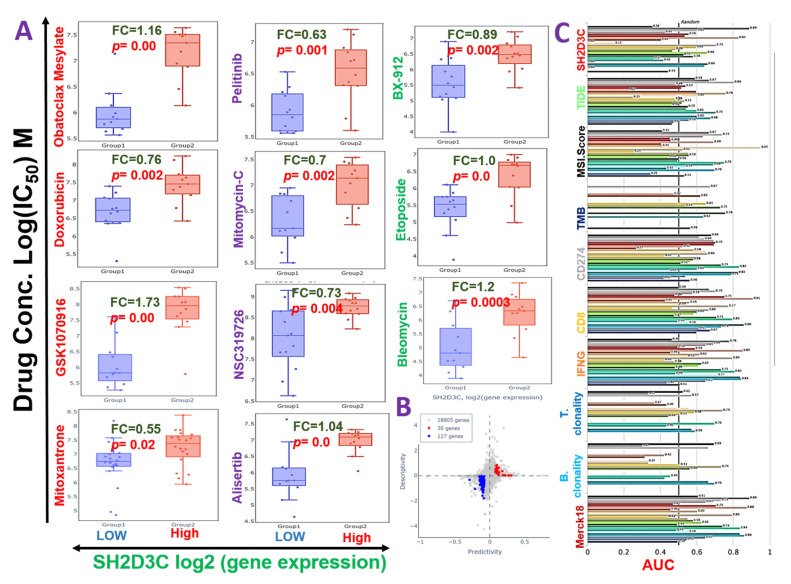
Non-structural protein 3 (*NSP3; SH2D3C*) is associated with chemotherapy resistance in NSCLC. (**A**) Bar plots showing the sensitivity of various clinical drugs between samples with low and high expression levels of *SH2D3C*. (**B**) Scatter plot of the efficacy of *SH2D3* knockdown (*shSH2D3C*) in association with the expression of a number gene. The predictivity is defined as the fold change (FC) of *shSH2D3C* efficacy between samples of high and low expression of the gene. Descriptivity is defined as the FC of gene expression between samples of high and low *shSH2D3C* efficacy. (**C**) Bar plot of the comparative biomarker relevance between the *SH2D3C* and standardized biomarkers.

We evaluated the cross-correlation between the expression levels of *SH2D3C* and the sensitivity of NSCLC cell lines to various clinical drugs. Our results revealed that high expression level of SH2D3C is associated with the resistance of NSCLC cell lines to doxorubicin, mitoxantrone, GSK1070916, obatoclax mesylate, alisertib, NSC319726, mitomycin-C, bleomycin, etoposide, pelitinib, and BX-912 (Figure 6A). Furthermore, we evaluated the efficacy of *SH2D3* knockdown (*shSH2D3C*) in association with the expression of a number of target genes. Interestingly, we found that higher expression levels of the target genes are associated with the low efficacy of *shSH2D3C* in NSCLC cell lines (Figure 6B). Analysis of the biomarker relevance revealed a clinical predictive value (AUC > 0.5) of SH2D3C in 12 datasets of immunotherapy cohorts. Interestingly, *SH2D3C* achieved a higher clinical predictive score in a larger number of datasets when compared with TMB, T-cell clonality, and B-cell clonality with a clinical predictive score in 7, 8, and 8 datasets of immunotherapy cohorts (Figure 6C). Summing up the results obtained so far, our study suggested that *SH2D3C* is an important player in immune and inflammatory events in the carcinogenesis of NSCLC. It is associated with response to therapy in NSCLC and thus serves as an attractive target for exploration.

### 3.7. The Organosulfur Compounds in *Allium sativum* Exhibited Desirable Physicochemical and Pharmacokinetics Properties of a Drug-Like Candidate

**Figure 7:**
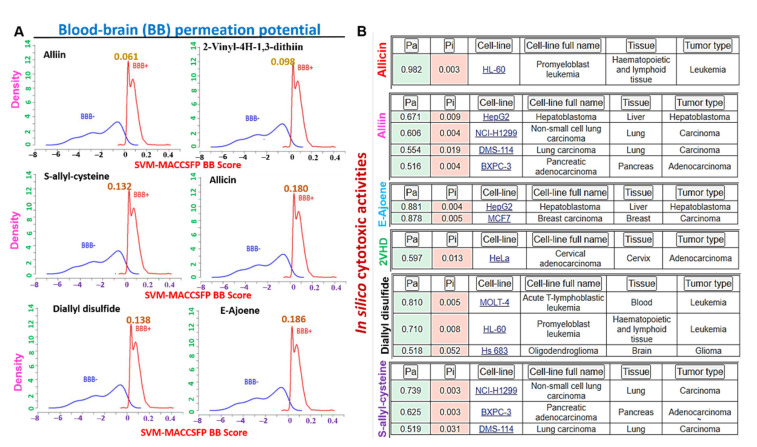
Blood−brain barrier permeation and in silico cytotoxic properties of some organosulfur compounds in garlic. (**A**) Blood−brain barrier penetration (BBB) permeation curve of some organosulfur compounds in *A. sativum*. The BBB permeation ability was measured by the support vector machine (SVM) and LiCABEDS algorithms of the BBB prediction server. (**B**) In silico cytotoxic properties of some organosulfur compounds in garlic against cancer cell lines. Pa = activity, pi = inactivity.

Our in silico analysis of the drug-likeness, physicochemical, and pharmacokinetic properties of the organosulfur small molecules from *A. sativum* (Table 1, Table S1, Figure S1) revealed good predictions of the six compounds for ADMET properties, drug-likeness, adherence to Lipinskís rules, and no alerts for PAINS (Table 1). Our analysis of the human gastrointestinal absorption and blood−brain barrier penetration ability of the compounds indicated that all the six compounds evaluated demonstrated BBB penetration ability (Figure 7A), with E-ajoene allicin and diallyl disulfide having higher brain-penetrant scores. QSAR modeling of acute toxicity in rats revealed estimated LD_50_ concentrations ranged between 74.190~937.700 mg/kg, 50.130~772.600 mg/kg BW, 429.00~3155.00 mg/kg, and 25.850~798.300 mg/kg for intraperitoneal, intravenous, oral, and subcutaneous route of administrations of the six compounds, respectively (Table 1), suggesting a high safety profile of the compounds especially when administered via the oral route.

### 3.8. The Organosulfur Compounds from *Allium sativum* Exhibited Selective in Silico Cytotoxic Activities against Cancer Cell Lines and Are not Cytotoxic to Normal Human Cell Lines

We used the in silico approach to evaluate the possible cytotoxic activities of E-ajoene, alliin, diallyl sulfide, 2-vinyl-4H-1,3-dithiin, allicin and **S**-allyl-cysteine on cancer cell lines as well as normal cell lines. Interestingly, we found that none of the compounds exhibit cytotoxic activities on the normal human cell lines; however, our results revealed a very strong cytotoxic activity (pa = 0.982) of allicin against leukemia cell line (HL-60), and strong activity of E-ajoene on the liver (pa = 0.881) and breast (pa = 0.878) cancer cell lines. Alliin was identified to be active against lung, liver, and pancreatic cancer cells (pa = 0.51~0.671). 2-vinyl-4H-1,3-dithiin demonstrated in silico cytotoxic activity against leukemia and brain cancer cell lines (pa = 0.518~0.810), while S-allyl-cysteine demonstrated activity against the lung and pancreatic cancer cell lines (pa = 0.519~0.739). Collectively, this study revealed that the six organosulfur compounds in garlic evaluated in this study exhibited selective cytotoxic activity on cancer cell lines and are not cytotoxic to normal human cell lines (Figure 7B).

### 3.9. Molecular Docking Profile of NSP3 (SH2D3C) with Some Organosulfur Compounds in *Allium Sativum*

**Figure 8:**
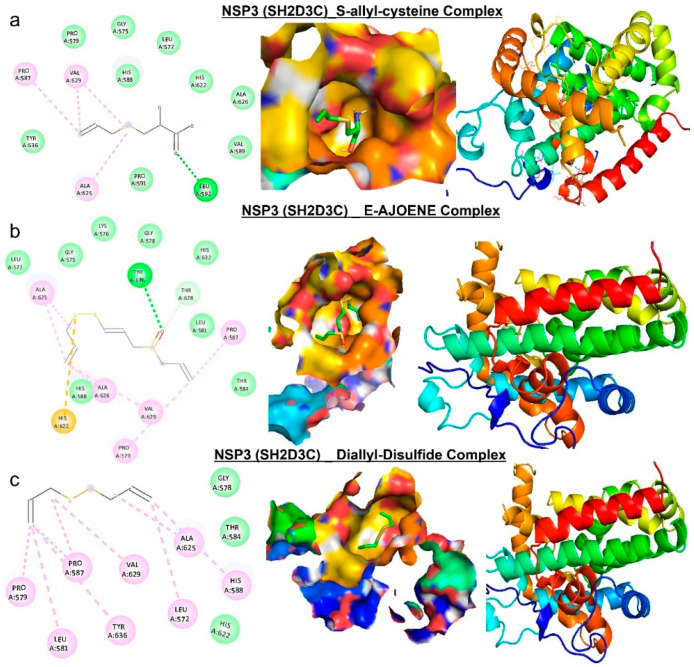
Molecular docking profile on *NSP3* with the organosulfur small molecule from *Allium sativum*. Two-dimensional (2D) structure and binding surface flip of the ligand−receptor interactions between *NSP3* (*SH2D3C*) and (**a**) S-allyl-cysteine, (**b**) E-ajoene, and (**c**) diallyl sulfide.

**Figure 9:**
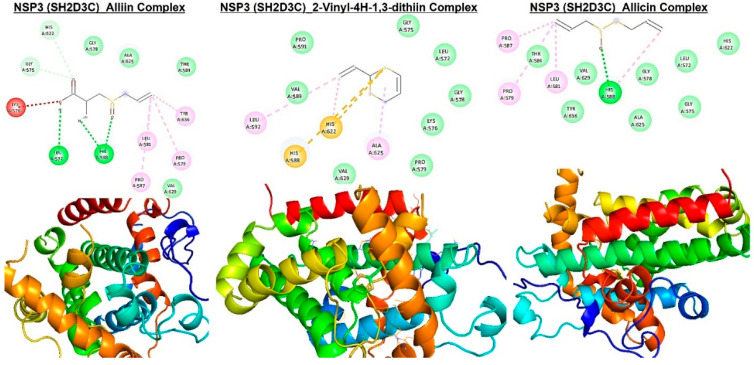
Molecular docking profile on novel SH2-containing protein 3 (*NSP3*) with the organosulfur small molecule from *Allium sativum*. Three- (3D) and two-dimensional (2D) structure of the ligand−receptor interactions between *NSP3* (*SH2D3C*) and alliin, allicin, and 2-vinyl-4H-1,3-dithiin.

Molecular docking studies revealed that E-ajoene, alliin, diallyl sulfide, 2-vinyl-4H-1,3-dithiin, and allicin docked well into the *NSP3 (SH2D3C)* binding cavity with binding affinities ranging −3.5~−6.70 Ă and random forest (RF) ranging 4.31~5.26 pKd (Table 2). The complexes are bounded by a number of conventional H-bonding and alkyl interactions. Furthermore, the complexes are stabilized by several hydrophobic contacts and van der Waals forces with several amino acids, surrounding the compound backbones in the receptor-binding pocket (Figure 8 and Figure 9).

## 4. Discussion

The impacts of cancer incidences and mortality are increasing at a global level. An estimated 19.3 million new cancer cases and almost 10.0 million cancer deaths occurred in 2020 [1]. Messenger (m)RNA expressions play important roles in cancer cell progression and survival, and comparative gene expression profiles between tumors and adjacent tissues have been very useful in identifying potential cancer biomarkers [65,66]. Our results suggest that *NSP3 (SH2D3C*) is differentially expressed in different cancer types and thus could serve different prognostic purposes in different cancer types. As such, different therapeutic approaches must be considered when targeting *NSP3 (SH2D3C)* in different cancer types. Controversial roles of proteins in different cancer types have been reported in previous studies; for instance, EPHB4 was described as an oncogene in some cancers and as a tumor suppressor in other cancers [67,68,69]. These contradictory findings suggest a complex role of *NSP3* signaling in cancer, characterized by multi-domain and multifunctional properties. Specifically, our differential expression and survival analyses strongly suggested that *NSP3 (SH2D3C*) is a potential biomarker of advanced stages of lung cancer.

Genes with similar expression patterns share similar functions. Therefore, gene functional enrichment analysis is important in an in silico approach and has been widely used in the analysis of gene-related pathological processes [70,71]. Our interaction network revealed a number of onco-functional genes including G3BP1, EIF4G1, PABPC1, BCAR1, CAPRIN1, EIF4A1, and EIF4A2, and HIRA as the functional partners associated with the immune-oncogenic roles of *SH2D3C*. Consequently, GO and KEGG pathway enrichment analyses were conducted to identify functional processes and pathways that may be mediated by these genes. Interestingly, our results suggested that *NSP3*-associated networks were involved in several pathways and biological processes associated with bacterial and viral infections, immune and inflammatory diseases, and cell proliferation and lung cancer development.

Tumor immune cell infiltrations are indicators of host immune responses to cancer cells [72,73]. Several studies revealed that some tumors have a high level of infiltration by cytotoxic T cells in a dysfunctional state and could not elicit tumor growth, while in other tumors, immunosuppressive factors may prevent T cells from infiltrating the tumors [74,75,76]. These immunosuppressive cells, including T reg cells, M2-TAMs, and CAFs, are known to promote tumorigenic and metastatic properties by inhibiting T-cell expansion, secreting cytokines, and remodeling the extracellular matrix (ECM) [77,78]. Interestingly, our analysis of gene expression correlations with tumor infiltrations of immune cells and immunosuppressive cells revealed that *NSP3* could regulate lung cancer tumor evasion of immune cells via both T-cell exclusion and induction of dysfunctional T-cell phenotypes. In addition, both genetic alterations and gene methylation of *NSP3* were also found to regulate dysfunctional T-cell phenotypes and are associated with the early death of lung cancer patients. Altogether, our findings strongly suggest that *NSP3* is an important onco-immunological biomarker encompassing the TME, disease staging, and prognosis in lung cancer, and can serve as an attractive target for cancer therapies. However, subsequent development of *NSP3* as a reliable therapeutic target in lung cancer requires preclinical and clinical validation.

Drug-likeness is a complex balance of molecular properties and structural features which determine whether a molecule is like known drugs [79,80]. Interestingly, our in silico analysis revealed that the six organosulfur compounds from *A. sativum* are good drug-like candidates and exhibited selective in silico cytotoxic activities against lung, brain, liver, and pancreatic cancers with no cytotoxic effects on normal cell lines. The compounds are BBB-penetrant and therefore will be very useful in treating glioblastomas and other diseases associated with the central nervous system (CNS). Evaluation of toxicity of natural products and active compounds is an important factor in the development of drugs for therapeutic applications [81,82,83]. Our in silico acute toxicity and ecotoxicity studies revealed that different routes of administration of the sulfur-containing compounds produced different LD_50_ levels in rats. The overall analysis, however, revealed that the oral route was the safest route for administering these compounds; this route should be well tolerated when used for acute administration and is currently being employed for in vivo evaluations of these compounds against lung cancer and glioblastomas in our laboratory.

Molecular docking has become an increasingly valuable tool for depicting the possible interaction between a small-molecule drug candidate and a protein target during the early stage of drug development [84,85,86]. Our molecular docking analysis indicated that the organosulfur compounds of *Allium sativum* have the molecular properties to interact efficiently with the binding site of *NSP3* and are currently under rigorous experimental validation in our laboratory. E-ajoene, alliin, diallyl sulfide, 2-vinyl-4H-1,3-dithiin, and allicin docked well into the binding cavity of *NSP3 (SH2D3C).* Hydrogen bonds and other noncovalent interactions, such as hydrophobic and ionic interactions and van der Waals forces, play important roles in stabilizing ligand−protein complexes [87]. We found that the interactions of the compounds with *NSP3* predominantly involved hydrogen bonds, van der Waals forces, π-alkyl, and various hydrophobic interactions. The van der Waals forces created around the compound’s backbone with the several amino acids create a strong cohesive environment, which further stabilized the complexes [88]. However, S-allyl-cysteine interaction with *NSP3 (SH2D3C)* is unfavorable and hence less susceptible to *NSP3* ligandability. Altogether, our molecular docking experiments suggested that the organosulfur compounds from *A. sativum* have the molecular properties to interact efficiently with the binding site of *NSP3*. This interaction is under rigorous experimental validation in in vitro as well as in vivo models of GBM and lung cancer.

## 5. Conclusions

In conclusion, our in silico study suggested that *NSP3* is an important onco-immunological biomarker encompassing the tumor microenvironment, disease staging, and prognoses in lung cancer and could serve as an attractive target for cancer therapies. Our molecular docking experiments suggested that the organosulfur compounds from *Allium sativum,* including alliin, allicin, E-ajoene, Z-ajoene, 2-vinyl-4H-1,3-dithiin, diallyl sulfide, and allyl methyl sulfide, have molecular properties allowing them to efficiently interact with the binding site of *NSP3* and are currently under rigorous experimental validation in our laboratory.

## Figures and Tables

**Table 1:** Drug likeness, medicinal chemistry, and physicochemical and ADMET properties of the organosulfur small molecules from *Allium sativum*.

Parameters	Alliin	Allicin	E-Ajoene	2-Vinyl-4H-1,3-Dithiin	Diallyl Sulfide	S-Allyl-Cysteine
Formula	C_6_H_11_NO_3_S	C_6_H_10_OS_2_	C_9_H_14_OS_3_	C_6_H_8_S_2_	C_6_H_10_S	C_4_H_8_S
M.W (g/mol)	177.22	162.27	234.40	144.26	114.21	88.17
Fraction Csp3	0.50	0.33	0.33	0.33	0.33	0.50
Num. rotatable bonds	5	5	8	1	4	2
Num. H-bond acceptors	4	1	1	0	0	0
Num. H-bond donors	2	0	0	0	0	0
Molar Refractivity	43.24	45.88	67.41	43.08	37.60	28.46
TPSA	99.60 Å^2^	61.58 Å^2^	86.88 Å^2^	50.60 Å^2^	25.30 Å^2^	25.30 Å^2^
Log *P*_o/w_ (XLOGP3)	−3.53	1.31	1.71	2.30	2.16	1.54
Log *S* (ESOL)	1.62	−1.34	−1.84	−2.12	−1.64	−1.21
Class	Highly soluble	Very soluble	Very soluble	Soluble	Very soluble	Very soluble
GI absorption	High	High	High	High	High	High
BBB permeant	No	Yes	No	Yes	Yes	Yes
Lipinski	Yes; 0 violation	Yes; 0 violation	Yes; 0 violation	Yes; 0 violation	Yes; 0 violation	Yes; 0 violation
Bioavailability score	0.55	0.55	0.55	0.55	0.55	0.55
Synthetic accessibility	3.21	3.60	4.33	3.91	2.34	1.92
	Acute toxicity					
IP LD_50_ (mg/kg)	347.700 (OECD:4)	77.750 (OECD:4)	74.190 (OECD:4)	31.610 (OECD:4)	937.700 (OECD:5)	162.90 (OECD:4)
IV LD_50_ (mg/kg)	772.600 (non-toxic)	54.520 (OECD:4)	141.600 (OECD:4)	66.460 (OECD:4)	50.130 (OECD:4)	61.150 (OECD:4)
Oral LD_50_ (mg/kg)	3155.00 (OECD:5)	468.200 (OECD:4)	1465.00 (OECD:4)	429.00 (OECD:4)	789.100 (OECD:4)	550.500 (OECD:4)
SC LD_50_ (mg/kg)	798.300 (OECD:4)	128.00 (OECD:4)	300.400 (OECD:4)	331.600 (OECD:4)	92.270 (OECD:3)	25.850 (OECD:4)
	Ecotoxicity					
Bioaccumulation factor Log10(BCF)	0.152	0.565	0.328	1.098	0.661	0.506
Daphnia magna LC_50_ -Log10(mol/L)	3.614	5.375	5.857	4.876	4.428	4.216
Fathead minnow LC50 Log10(mmol/L)	−0.173	−1878	−2.122	−2.189	−1.902	−1.300
Tetrahymena pyriformis IGC50 -Log10(mol/L)	−0.597	0.803	1.131	1.091	0.425	0.128

**Table 2:** Docking profile of *NSP3* (*SH2D3C*) with the organosulfur small molecule from *Allium sativum*.

	S-allyl-cysteine	E-AJOENE	Alliin	Diallyl-Disulfide	Allicin	2-Vinyl-4H-1,3-dithiin
ΔG (Kcal/mol)	−4.40	−4.40	−6.70 −4.70	−3.50	−3.90	−4.00
Hydrophobic contact	PRO587 (3.85 Ă) HIS622A (3.78 Ă) ILE131 (3.62 Ă) PHE132 (3.61 Ă) PHE156 (3.70 Ă)	PRO579 (3.50), LEU581 (3.52), THR584 (3.56), PRO587 (3.79), ALA625 (3.62)	Pro579 (3.99), leu581 (3.89), thr584 (3.78), pro587 (3.94)	Leu572 (3.72), pro579 (3.90), leu581 (3.79), thr584 (3.67), pro587 (3.79), his588 (3.75)	Leu572 (3.87), pro579 (3.92), leu581 (3.77), thr584 (3.89), pro587 (3.51), his588 (3.74)	His588 (3.75), his622 (3.76), ala625 (3.53)
Conventional H-bond	LEU592 (2.21)	Thr636 (2.61) Thr628 (3.64)	His622, gly575, leu572, his588		His588	
Pi-sulfur		his622				His588, his622
alkyl interaction	VAL626, PRO587 ALA625	Ala625, ala626, val629, pro579, pro587	Tyr636, leu581, pro587, pro579	Pro579, pro587, val629, ala625, leu581, tyr636, leu572, his588	Pro587, pro579, leu581	Leu592, ala625
Van der waal forces	Pro579, Gly575, His588, Leu572, His622, Ala626, Val589, Pro591, Tyr636	Leu572, Gly585, Lys576, Gly578, His632, Leu581, Thr584, His 588	Val629, Thr584, Ala625, Gly578	His622, Gly578	Thr584, Tyr636, Val629, Ala626, Gly578, Gly575, Leu572, His622	Pro591, Val589, Val629, Pro579, Lys576, Gly578, Leu572, Gly575

## Data Availability

Not applicable.

## Supplementary Materials

The following are available online at https://www.mdpi.com/2227-9059/9/11/1582/s1, Figure S1: The bioavailability radar of organosulfur compounds from *Allium sativum*; Table S1: The chemical structures of the main organosulfur compounds in garlic.
