# Correction: Yeh et al. Identification of *NSP3* (*SH2D3C*) as a Prognostic Biomarker of Tumor Progression and Immune Evasion for Lung Cancer and Evaluation of Organosulfur Compounds from *Allium sativum* L. as Therapeutic Candidates. *Biomedicines* 2021, *9*, 1582

**DOI:** 10.3390/biomedicines13010188

**Published:** 2025-01-14

**Authors:** Yuan-Chieh Yeh, Bashir Lawal, Michael Hsiao, Tse-Hung Huang, Chi-Ying F. Huang

**Affiliations:** 1Program in Molecular Medicine, College of Life Sciences, National Yang Ming Chiao Tung University, Taipei 11221, Taiwan; b9005030@gmail.com; 2Department of Traditional Chinese Medicine, Chang Gung Memorial Hospital, Keelung 20401, Taiwan; 3PhD Program for Cancer Molecular Biology and Drug Discovery, College of Medical Science and Technology, Taipei Medical University, Taipei 11031, Taiwan; bashirlawal12@gmail.com; 4Graduate Institute of Cancer Biology & Drug Discovery, College of Medical Science and Technology, Taipei Medical University, Taipei 11031, Taiwan; 5Genomics Research Center, Academia Sinica, Taipei 115201, Taiwan; mhsiao@gate.sinica.edu.tw; 6School of Traditional Chinese Medicine, Chang Gung University, Kweishan, Taoyuan 333, Taiwan; 7School of Nursing, National Taipei University of Nursing and Health Sciences, Taipei 112, Taiwan; 8Graduate Institute of Health Industry Technology, Chang Gung University of Science and Technology, Taoyuan 333, Taiwan; 9Research Center for Chinese Herbal Medicine, Chang Gung University of Science and Technology, Taoyuan 333, Taiwan; 10Department & Graduate Institute of Chemical Engineering & Graduate Institute of Biochemical Engineering, Ming Chi University of Technology, New Taipei City 243, Taiwan; 11Institute of Biopharmaceutical Sciences, College of Pharmaceutical Sciences, National Yang Ming Chiao Tung University, Taipei 11221, Taiwan; 12Department of Biochemistry, School of Medicine, Kaohsiung Medical University, Kaohsiung 80708, Taiwan

## Error in Figures/Table

In the original publication [1], there were mistakes in Figures 8 and 9 and Table 2 as published. The mistake involves the use of “Non-structural protein 3 (*NSP3*)” instead of “novel SH2-containing protein 3 (*NSP3*)”. The corrected Figure 8 and Figure 9 and Table 2 appears below.

**Figure 8 biomedicines-13-00188-f008:**
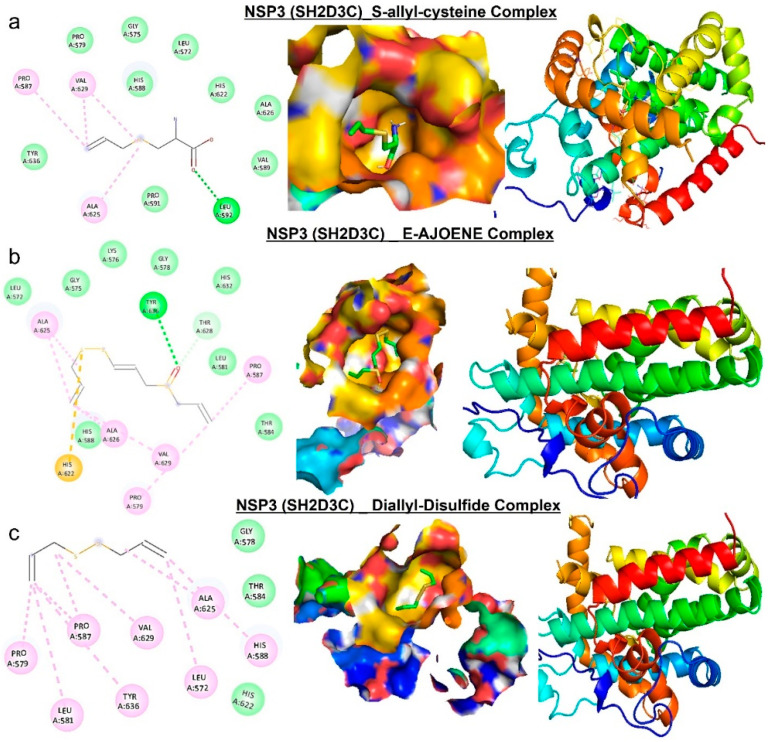
Molecular docking profile on *NSP3* with the organosulfur small molecule from *Allium sativum.* Two-dimensional (2D) structure and binding surface flip of the ligand−receptor interactions between *NSP3* (*SH2D3C*) and (**a**) S-allyl-cysteine, (**b**) E-ajoene, and (**c**) diallyl sulfide.

**Figure 9 biomedicines-13-00188-f009:**
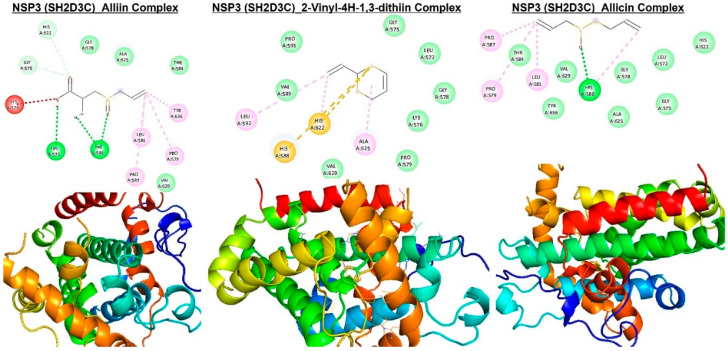
Molecular docking profile on novel SH2-containing protein 3 (*NSP3*) with the organosulfur small molecule from *Allium sativum.* Three- (3D) and two-dimensional (2D) structure of the ligand−receptor interactions between *NSP3* (*SH2D3C*) and alliin, allicin, and 2-vinyl-4H-1,3-dithiin.

**Table 2 biomedicines-13-00188-t002:** Docking profile of *NSP3* (*SH2D3C*) with the organosulfur small molecule from *Allium sativum*.

	S-allyl-cysteine	E-AJOENE	Alliin	Diallyl-Disulfide	Allicin	2-Vinyl-4H-1,3-dithiin
ΔG (Kcal/mol)	−4.40	−4.40	−6.70 −4.70	−3.50	−3.90	−4.00
Hydrophobic contact	PRO587 (3.85 Ӑ) HIS622A (3.78 Ӑ) ILE131 (3.62 Ӑ) PHE132 (3.61 Ӑ) PHE156 (3.70 Ӑ)	PRO579 (3.50), LEU581 (3.52), THR584 (3.56), PRO587 (3.79), ALA625 (3.62)	Pro579 (3.99), leu581 (3.89), thr584 (3.78), pro587 (3.94)	Leu572 (3.72), pro579 (3.90), leu581 (3.79), thr584 (3.67), pro587 (3.79), his588 (3.75)	Leu572 (3.87), pro579 (3.92), leu581 (3.77), thr584 (3.89), pro587 (3.51), his588 (3.74)	His588 (3.75), his622 (3.76), ala625 (3.53)
Conventional H-bond	LEU592 (2.21)	Thr636 (2.61) Thr628 (3.64)	His622, gly575, leu572, his588		His588	
Pi-sulfur		his622				His588, his622
alkyl interaction	VAL626, PRO587, ALA625	Ala625, ala626, val629, pro579, pro587	Tyr636, leu581, pro587, pro579	Pro579, pro587, val629, ala625, leu581, tyr636, leu572, his588	Pro587, pro579, leu581	Leu592, ala625
Van der waal forces	Pro579, Gly575, His588, Leu572, His622, Ala626, Val589, Pro591, Tyr636	Leu572, Gly585, Lys576, Gly578, His632, Leu581, Thr584, His 588	Val629, Thr584, Ala625, Gly578	His622, Gly578	Thr584, Tyr636, Val629, Ala626, Gly578, Gly575,Leu572, His622	Pro591, Val589, Val629, Pro579, Lys576, Gly578, Leu572, Gly575

## Text Correction

There were errors in the original publication. The mistake involves the use of “Non-structural protein 3 (*NSP3*)” instead of “novel SH2-containing protein 3 (*NSP3*)”.

A correction has been made to *Abstract*:

(1) Original: The multi-domain non-structural protein 3 (*NSP3*) is an oncogenic molecule that has been concomitantly implicated in the progression of coronavirus infection.

Revised: The novel SH2-containing protein 3 (*NSP3*) is an oncogenic molecule that has been concomitantly associated with T cell trafficking.

(2) Original: −4.3~−6.70 Ă

Revised: −3.5~−6.70 Ă

(3) Removed “However, S-allyl-cysteine interaction with *NSP3* (*SH2D3C*) is unfavorable and hence less susceptible to *NSP3* ligandability.”

A correction has been made to *Result*, *Section 3.9*:

Removed: However, S-allyl-cysteine interaction with *NSP3* (*SH2D3C*) is unfavorable (Figure 8A) and hence the least ΔG (−4.30 Kcal/mol) and RF (−4.31 pKd) values, respectively.

A correction has been made to *Discussion*, *the first Paragraph*:

Removed: Notwithstanding, *NSP3* was proposed to be a promising therapeutic target in both cancer and COVID-19.

A correction has been made to *Introduction*, *the third Paragraph*:

Original: Non-structural protein 3 (*NSP3*) is a multi-domain, multifunctional protein that is an essential component of the replication/transcription complex (RTC), responsible for the synthesis and processing of RNA, and interference with the innate immune system of host cells [20,21]. *NSP3* has been implicated in cancer progression and metastasis [22,23]. It is a key component in coronavirus replication and has thus played a pivotal role in the coronavirus disease 2019 (COVID-19) pandemic. It thus could be an attractive target for the development of therapeutic strategies for treating cancer and coronavirus infections [24].

Revised: The novel SH2-containing protein 3 (*NSP3*) is an oncogenic molecule that regulates T cell receptor signaling [20]. It acts as an adapter protein that mediates cell signaling pathways involved in cellular functions such as cell adhesion and migration, tissue organization, and the regulation of the immune response [20–22]. It thus could be an attractive target for the development of therapeutic strategies for treating cancer.

## References

Remove References [20–23] from the original manuscript [1] and use References [2,3,4] as [20–22]. With this correction, the order of some references has been adjusted accordingly.

The authors state that the scientific conclusions are unaffected. This correction was approved by the Academic Editor. The original publication has also been updated.
