# Supplementary material for: Identification of NSP3 (SH2D3C) as a Prognostic Biomarker of Tumor Progression and Immune Evasion for Lung Cancer and Evaluation of Organosulfur Compounds from Allium sativum L. as Therapeutic Candidates
Source: Biomedicines. 2021 Oct 30;9(11):1582. doi: 10.3390/biomedicines9111582 (PMC8615911; doi:10.3390/biomedicines9111582)
Supplement: Supplementary file 1 [file biomedicines-09-01582-s001.zip › biomedicines-1416042-supplementary.pdf]

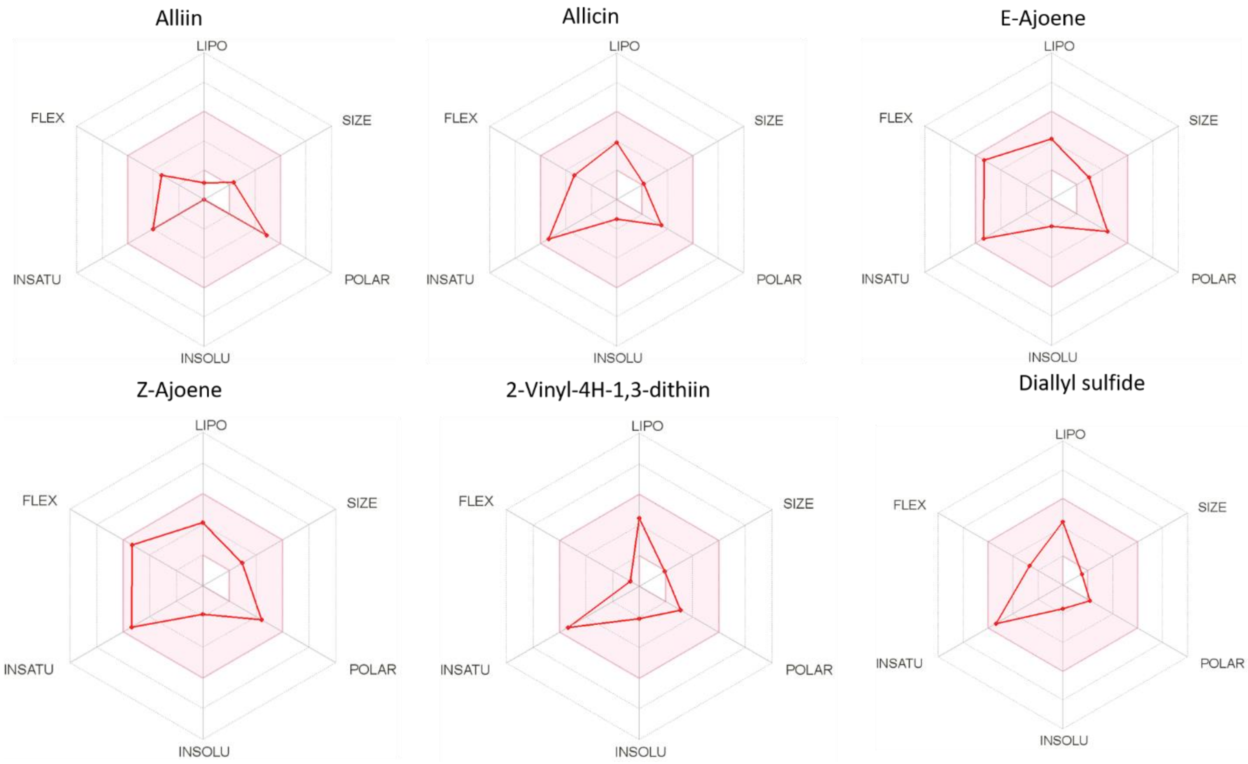

Figure S1. The bioavailability radar of E-Ajoene, alliin, Diallyl sulfide, Z-Ajoene, 2-Vinyl-4H-1,3-dithiin and Allicin. All the compounds evaluated fall within the pink area of the hexagon, which represents the optimal range for lipophilicity, polarity, solubility, saturation, and flexibility of a good drug like candidate.

Table S1: The chemical structures of the main organosulfur compounds in garlic.

Compounds	Molecular Formular	MW (g/mol)	IUPAC Name	Canonical SMILES
Alliin	C6H11NO3S	177.22	(2R)-2-amino-3-prop-2-enylsulfinylpropanoic acid	C=CCS(=O)CC(C(=O)O)N
Allicin	C6H10OS2	162.3	3-prop-2-enylsulfinylsulfanylprop-1-ene	C=CCSS(=O)CC=C
E-Ajoene	C9H14OS3	234.4	(E)-1-(prop-2-enyldisulfanyl)-3-prop-2-enylsulfinylprop-1-ene	C=CCSSC=CCS(=O)CC=C
Z-Ajoene	C9H14OS3	234.4	(Z)-1-(prop-2-enyldisulfanyl)-3-prop-2-enylsulfinylprop-1-ene	C=CCSSC=CCS(=O)CC=C
2-Vinyl-4H-1,3-dithiin	C6H8S2	144.3	2-ethenyl-4H-1,3-dithiine	C=CC1SCC=CS1
Diallyl sulfide	C6H10S	114.21	3-prop-2-enylsulfanylprop-1-ene	C=CCSCC=C
Diallyl disulfide	C6H10S2	146.3	3-(prop-2-enyldisulfanyl)prop-1-ene	C=CCSSCC=C
Diallyl trisulfide	C6H10S3	178.3	3-(prop-2-enyltrisulfanyl)prop-1-ene	C=CCSSSCC=C
Allyl methyl sulfide	C4H8S	88.17	3-methylsulfanylprop-1-ene	CSCC=C
S-allyl-cysteine	C6H11NO2S	161.2	(2R)-2-amino-3-prop-2-enylsulfanylpropanoic acid	C=CCSCC(C(=O)O)N
